# First report of *Cytauxzoon* sp. infection in a domestic cat from Portugal

**DOI:** 10.1186/s13071-016-1506-5

**Published:** 2016-05-10

**Authors:** Ana Margarida Alho, Joana Silva, Maria João Fonseca, Filipa Santos, Cláudia Nunes, Luís Madeira de Carvalho, Manuel Rodrigues, Luís Cardoso

**Affiliations:** CIISA, Faculty of Veterinary Medicine, Universidade de Lisboa (ULisboa), Lisbon, Portugal; Biopremier S.A., Campus da Faculdade de Ciências, ULisboa, Lisbon, Portugal; Hospital do Gato, Lisbon, Portugal; Department of Veterinary Sciences, School of Agrarian and Veterinary Sciences, University of Trás-os-Montes e Alto Douro (UTAD), Vila Real, Portugal

**Keywords:** *Cytauxzoon* sp., *Cytauxzoon manul*, Haemoparasite, Piroplasm, Domestic cat, Tick-borne disease, Portugal

## Abstract

**Background:**

Cytauxzoonosis is an emerging and life-threatening tick-borne feline disease caused by haemoprotozoan parasites of the genus *Cytauxzoon*. Information regarding epidemiological and clinical presentation of infections by species other than *Cytauxzoon felis* is scant. A case of *Cytauxzoon* sp. infection is described in a 2-year-old mixed breed male domestic cat from Portugal, presenting a history of acute lethargy, anorexia and pyrexia.

**Results:**

Complete blood count revealed a severe anaemia, leucocytosis and thrombocytopenia. A pleural effusion was noticed on thoracic radiograph, and marked splenomegaly and free abdominal fluid were visualized by ultrasound. A molecular screening for the detection of causative agents of infectious anaemia was performed, and a positive result for Piroplasmorida was obtained. DNA sequencing of a 743 bp amplicon of the 18S rRNA gene (GenBank accession no. KU710344) revealed 99.9 % identity with *Cytauxzoon manul*.

**Conclusions:**

This is the first report of *Cytauxzoon* sp. (clustering together with *C. manul*) in a felid from Portugal. Clinical manifestations along with molecular analysis suggest the hypothesis that domestic cats might be infected with and serve as a reservoir host for *C. manul*.

## Background

Feline vector-borne diseases are being increasingly reported worldwide. Several factors have been linked to this sharp expansion and wide distribution range, namely climate changes, enhanced international commerce and global transport, increased drug resistance among vectors and pathogens, demographic and political changes, and wildlife host abundance [1, 2].

Cytauxzoonosis is an emerging tick-borne feline disease caused by haemoprotozoan parasites of the genus *Cytauxzoon* (Theileriidae), with a few identified species [3]. This life-threatening pathological condition is characterized by a rapid course of illness and eventually death, usually in a couple of days. *Cytauxzoon felis* is the main agent of cytauxzoonosis, with different strains or genotypes capable of producing infection in domestic cats, lions and tigers [4, 5]. A closely related piroplasm was reported in Pallas’s cats (*Otocolobus manul*) from Mongolia and later described as a new species, *Cytauxzoon manul*, based on a significant sequence divergence [6, 7]. Furthermore, *C. manul* has also been reported in African lions [8]; and *Cytauxzoon* sp. clustering together with *C. manul* in domestic cats [9–11], an Iberian lynx [12, 13], Eurasian lynxes and wildcats [14]. Experimental infection of domestic cats with blood from Pallas’s cats infected with *C. manul* showed that they are susceptible to erythrocytic phases of this agent and presumably to other phases of the parasite’s life-cycle [15]. Nevertheless, information regarding epidemiological distribution, clinical presentation, genetics and pathogenicity of infection by *C. manul* is scant. Besides, very little is known about species of *Cytauxzoon* other than *C. felis,* especially in Europe. Here, we report the first clinical case and molecular characterization of naturally occurring *Cytauxzoon* sp. infection in a domestic cat (*Felis catus*) from Portugal.

## Clinical case

In February 2015, a 2-year-old mixed breed intact male domestic cat (body condition score of 5/9) was presented to a referral veterinary hospital in Lisbon, with a history of acute onset of lethargy, anorexia and pyrexia. The cat was born and raised in the northern central region of Portugal) and had never travelled abroad. The animal had regular outdoor access and shared the house and backyard with its siblings. No health issues had previously been diagnosed, and its past history was unremarkable.

At physical examination the cat was severely depressed and had tachycardia, dyspnoea and tachypnoea. Body temperature was 40.0 °C. Mucous membranes were pale and capillary refill time was > 2 s. Blood was collected for a complete blood count (CBC), a serum chemistry profile and rapid tests for the detection of feline leukaemia virus (FeLV) antigen and of antibodies to feline immunodeficiency virus (FIV). CBC revealed a severe anaemia, leucocytosis and thrombocytopenia and routine serum biochemistry showed azotaemia and hyperbilirubinemia (Table 1). FIV and FeLV tests were negative.

**Table 1:** Complete blood count (CBC) and routine serum biochemistry results

Parameter	Values (unit)	Reference values (unit)
Platelet count	85 × 10^3^/mm^3^	150–500 × 10^3^/mm^3^
Red blood cell count	3.14 × 10^6^/mm^3^	5.0–11.0 × 10^6^/mm^3^
White blood cell count	19.6 × 10^3^/mm^3^	5.5–19.5 × 10^3^/mm^3^
Alanine transaminase (ALT)	46 U/l	20–100 U/l
Albumin	2.6 g/dl	2.2–4.4 g/dl
Alkaline phosphatase (ALP)	27 U/l	10–90 U/l
Bilirubin	0.7 mg/dl	0.1–0.6 mg/dl
Blood urea nitrogen (BUN)	66 mg/dl	10–30 mg/dl
Chloride	123 mmol/l	107–120 mmol/l
Creatinine	1.5 mg/dl	0.3–2.1 mg/dl
Globulin	3.7 g/dl	1.5–5.7 g/dl
Glucose	150 mg/dl	70–150 mg/dl
Haematocrit	16.0 %	24–45 %
Haemoglobin	4.9 g/dl	8.0–15.0 g/dl
Potassium	3.9 mmol/l	3.4–4.6 mmol/l
Sodium	151 mmol/l	147–156 mmol/l
Total proteins	6.3 g/dl	5.4–8.2 g/dl

To assess the cause of the dyspnoea and tachypnoea, lateral and ventrodorsal radiographic projections of the thorax were performed at full inspiration, revealing pleural effusion. Further abdominal ultrasound showed marked splenomegaly, kidneys with loss of definition and marked amount of anechoic (low cellularity) free fluid in the abdomen. Pleural and abdominal liquids collected and sent for cytology were compatible with a non-septic exudate (mixed cell types, moderate cellularity, predominance of non-degenerate neutrophils without phagocytized bacteria, presence of foamy macrophages, mature lymphocytes and, occasionally, reactive mesothelial cells), probably as a consequence of an increased permeability secondary to inflammation and vascular damage.

The cat was hospitalized and started oxygen therapy. Additionally, intravenous (IV) crystalloid fluid therapy with potassium chloride was given to correct the dehydration and provide fluid therapy maintenance. Antibiotic treatment with ceftriaxone (25 mg/kg, IV, twice a day [BID]) along with doxycycline (10 mg/kg, orally [PO], once a day [SID], for 21 days) was started, and midazolam (0.2 mg/kg, intramuscularly [IM], SID) was given to increase appetite. Due to the low haematocrit and the low haemoglobin concentration, a red cell concentrate was administered to increase the supply of oxygen to the tissues.

Whole blood in EDTA was molecularly screened for agents of infectious feline anaemia. DNA was extracted with High Pure PCR Template Preparation Kit (Roche Diagnostics GmbH, Germany). A PCR with primers Mycop F1 and Mycop Rev1 for detection of Mycoplasmatales, including the genus *Mycoplasma* (Portuguese Institute for Accreditation [IPAC] accredited test ref. PT03.12; Biopremier, Portugal), yielded a negative result. The amplification program was as follows: an initial step at 94 °C for 2 min, 45 cycles of 30 s at 94 °C, 30 s at 57 °C and 1 min at 72 °C, and final extension at 72 °C for 5 min. The PCR kit HaemoTicks (ref. BIOV IA 48; Biopremier, Portugal) was used for the detection of Rickettsiales (including the genera *Anaplasma*, *Ehrlichia* and *Rickettsia*), with primers RickO F2 and RickO Rev1; and of Piroplasmorida (comprising the genera *Babesia*, *Cytauxzoon* and *Theileria*), with primers Piro F2 and Piro Rev2. The amplification program was as follows: an initial step at 94 °C for 2 min, 40 cycles of 30 s at 94 °C, 30 s at 58.5 °C and 15 s at 72 °C, and final extension at 72 °C for 5 min. A negative result was obtained for Rickettsiales; and a positive result for Piroplasmorida. An amplicon with 743 bp of the 18S rRNA gene was sequenced (GenBank accession no. KU710344). A BLAST analysis of the GenBank database (http://www.ncbi.nlm.nih.gov/BLAST/) revealed 99.9 % identity with the only two DNA sequences of *C. manul* available (accession nos. AY485690 and AY485691; corresponding to 1 nucleotide substitution), from two Pallas’s cats caught in Mongolia [5]; 100 % identity with sequences of *Cytauxzoon* sp. (GenBank accession nos. HM146422 and HM146424) from domestic cats sampled in Italy [11]; 99.9 % identity with sequences of *Cytauxzoon* sp. (GenBank accession nos. AY309956 and EU622908) from domestic cats sampled in Spain [9] and France [10], respectively; and 100 % identity with several other sequences from wild mammals deposited in GenBank also as *Cytauxzoon* sp., including: AY496273, from an Iberian lynx (*Lynx pardinus*) sampled in Spain [12]; KT361074, from a wildcat (*Felis silvestris*) sampled in Romania [14]; and KT361080, from a Eurasian lynx also sampled in Romania [14]. The maximum homology obtained with the second closest species, *C. felis*, was 96.7 % (corresponding to 23 nucleotide substitutions).

**Fig. 1:**
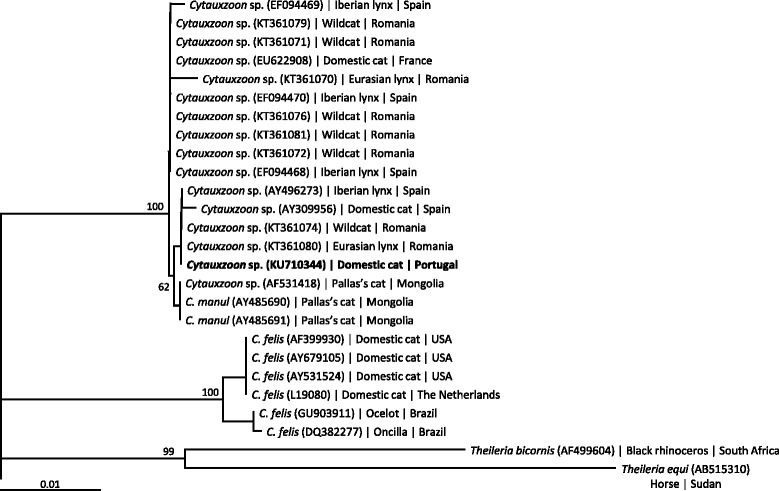
Phylogenetic tree of the 18S rRNA gene sequences of *Cytauxzoon* spp. The method of HKY85 was applied for calculating distances, and tree topology was inferred by neighbour-joining (Paup 4.0b10). Numbers at the nodes indicate bootstrap support obtained in 1,000 replications. *Theileria bicornis* and *Theileria equi* were used as outgroup. The new sequence is written in bold

A phylogenetic analysis including the DNA sequence obtained in the present study was performed to compare it with other sequences of *Cytauxzoon* spp. previously deposited in GenBank. Sequences were aligned with Clustal X version 1.83 (http://www.clustal.org/). A phylogenetic tree (Fig. 1) was computed with PAUP version 4.0b10 (http://paup.csit.fsu.edu/) using the HKY85 model [16] for calculating distances, and neighbour-joining method with 1,000 replications to estimate the node reliability. Gaps were treated as missing data.

Giemsa-stained blood smears were prepared from peripheral blood to check for intraerythrocytic piroplasms by using an ×1,000 magnification, but erythroparasitaemia was not observed. During the subsequent days, the cat’s body temperature remained high and haematocrit dropped to 7.6 %. After the molecular confirmation of *Cytauxzoon* sp., the animal was treated with azithromycin (10 mg/kg, SID, for 10 days), as atovaquone was not available. Despite daily monitoring and intensive supportive care, the cat’s condition declined over the following ten days and the animal died. No post-mortem pathological examination was feasible.

## Discussion

To the authors’ best knowledge, this is the first report of infection with *Cytauxzoon* sp. (clustering together with *C. manul*) in a felid from Portugal, as demonstrated by molecular analysis. Previous studies have reported cases of infection with *Cytauxzoon* sp. in domestic cats from southern Europe, namely Spain, France and Italy [9–11]. In these cases, the detected agent *Cytauxzoon* sp., although not named as such, was molecularly identical to *C. manul*. The study by Carli et al. [11] reported clinical disease and persistent infection in Italian cats, but most animals out of 115 cats were subclinically infected.

Previously considered a rare disease, cytauxzoonosis due to *C. felis* has now been reported in more than one third of the United States (especially in south-eastern and south-central states) and also in South America and in Europe, with an expected increase in the geographical range due to the ubiquity of tick vectors and their high capacity to adapt to different environments and host species [17–19]. *Cytauxzoon manul* was reported in Pallas’s cats from Mongolia [7] and in captive lions (*Panthera leo*) in Zimbabwe [8]; and *Cytauxzoon* sp./*C. manul* in Eurasian lynxes and wildcats from Romania [14]. Natural infection in domestic cats by *C. manul* might have involved a species jump from Pallas’s cats.

Cytauxzoonosis is usually associated with an outdoor exposure, particularly to unhewn rural areas, where contact with ticks is more frequent [11, 19]. The most common clinical findings of cytauxzoonosis are anorexia, depression, anaemia, vomiting, icterus and high fever [20]. Pancytopenia, splenomegaly and hepatomegaly are also common. However, there are no pathognomonic findings on CBC, serum chemistry, urinalysis or imaging studies [19]. Such non-specific and wide variety of clinical signs makes it impossible to confirm or exclude this diagnosis based exclusively on a clinical assessment. A conclusive diagnosis can be attained through the visualization of reticuloendothelial cells packed with *Cytauxzoon* schizonts (basophilic and amorphous protozoal bodies in the cell cytoplasm). Likewise, the visualization of intraerythrocytic piroplasms, i.e. 1–2 μm organisms with light blue cytoplasm and a dark red nucleus (commonly described as “signet ring”) in Giemsa-stained peripheral blood smears might help the diagnosis. Confirmation can be done with a more sensitive and specific method, like the PCR, even though it cannot be used to differentiate between acute and chronic cytauxzoonosis. Nevertheless, due to the extremely rapid course of illness associated with this disease, usually with no specific physical findings, a diagnosis is often made only by post-mortem examination. In the present case, the cat was presented with acute lethargy, anorexia and pyrexia, and the rapid course of illness led to death in a few days, with a confirmation of *Cytauxzoon* sp. by molecular analysis. In previous experimental infection with *C. manul* in domestic cats there was a low parasitaemia, but clinical signs were absent [15]. In the present report, considering the severity of clinical signs, the absence of immunosuppression factors and young age of the cat, we presume that *Cytauxzoon* sp. clustering together with *C. manul* might be highly virulent in domestic cats.

Cytauxzoonosis by *C. felis* is estimated to cause death in 90 % of the infected cats [20]. For that reason, treatment should be started as soon as possible for all cats clinically suspected of cytauxzoonosis, even if a definitive diagnosis has not been confirmed [19]. The standard of care for cytauxzoonosis appears to be a 10-day course of the antimalarial atovaquone (15 mg/kg, PO, three times a day [TID]) combined with the antibiotic azithromycin (10 mg/kg, PO, SID), along with an aggressive supportive therapy for sepsis [21]. However, atovaquone remains an expensive drug, which many owners are not able to afford, and furthermore is hard to get. Even with the recommended therapy, some cats remain persistent carriers of *C. felis*, serving as reservoirs for infection via a tick vector [19]. A recommended therapy could not be found specifically for *C. manul* in the scientific literature.

*Amblyomma americanum*, the lone star tick, is a highly competent vector for *C. felis* [22]. *Dermacentor variabilis* has also been experimentally demonstrated as a vector [23]. As these ticks have so far not been described in Portugal, *Rhipicephalus sanguineus* (*sensu lato*), *Ixodes* spp. or *Dermacentor* spp. might be hypothesized as being involved in the transmission of *Cytauxzoon* sp. in the country. Current preventive strategies in regions of endemicity are limited to prophylactic tick control (fipronil in spot-on formulations or imidacloprid/flumethrin in collars) and keeping cats indoors, in order to reduce exposure to ectoparasites and their transmitted pathogens [24, 25].

## Conclusions

Clinical manifestations along with the molecular analysis support the assumption that domestic cats might be infected with and serve as a reservoir host for *Cytauxzoon* sp. clustering together with *C. manul*. It is also suggested that interspecies transmission might be more frequent than previously thought. Further studies are needed to improve scientific knowledge on the biology and genetic diversity of this parasite, especially including its vectors, its vertebrate hosts and their geographical range. This report will increase the focus of the veterinary medical community towards *C. manul* in domestic cats.

### Ethical approval

All the clinical procedures in this study were in accordance with the Portuguese legislation for the protection of animals (Decree-Law n° 113/2013), as ascertained by the board of Hospital do Gato.
